# Protective effects of *Indigofera argentea* against acetic acid-induced inflammatory bowel disease are mediated by modulating antioxidant and inflammatory mediators 

**DOI:** 10.22038/AJP.2024.24977

**Published:** 2025

**Authors:** Faraza Javaid, Qaiser Jabeen

**Affiliations:** 1 *Department of Pharmacology, Faculty of Pharmacy, The Islamia University of Bahawalpur, Pakistan*; 2 *College of Pharmacy, Quaid-e-Azam Educational Complex, Sahiwal, Pakistan*

**Keywords:** Acetic acid, Cytokines, Reactive oxygen species, Ulcerative colitis

## Abstract

**Objective:**

The plant *Indigofera argentea *Burm. f., belonging to the family Fabaceae, is locally known as Hathio, Jantar and Neel. This plant is commonly found in deserted habitat and used traditionally to treat inflammatory and gastric disorders. The current study was designed to evaluate the phytochemical profile, toxicity and intestinal anti-inflammatory effects of the whole plant crude extract against acetic acid-induced inflammatory bowel disease (IBD) in mice.

**Materials and Methods:**

To study the phytochemical composition, preliminary phytochemical analysis along with HPLC and LCMS of the crude extract of *Indigofera argentea* (Ia.Cr) was performed. For the evaluation of intestinal anti-inflammatory activity, animals were divided into five groups (normal control, intoxicated, standard and two treatment groups) of six animals each and ulcerative colitis (UC) was induced by intrarectal administration of acetic acid (200 µl of 7.5%) and extent of damage caused by ulcerative colitis was measured by colonic mucosal damage index, disease activity index, and hematological and histological changes. Lipo-peroxide activity by malondialdehyde (MDA) and antioxidant enzyme glutathione peroxidase (GPX-1), catalase (CAT) and superoxide dismutase (SOD) levels were determined in colon tissues. Pro-inflammatory cytokines (IL-1, IL-6 and TNF-α) were quantified by ELISA immunoassay.

**Results:**

Pre-treatment with Ia.Cr significantly amended macroscopic damage, and hematological and histopathological alterations. Ia.Cr decreased oxidative parameters such as MDA and increased antioxidant activities of GPX-1, CAT and SOD. Ia.Cr also decreased the levels of pro-inflammatory biomarkers significantly.

**Conclusion:**

Results of this study indicated that Ia.Cr protected mice against acetic acid-induced inflammatory bowel disease by decreasing endogenous inflammatory biomarkers and oxidative damages.

## Introduction

Inflammatory bowel disease (IBD) is a group of global diseases that affect life quality and work capacity of individuals. Inflammatory bowel diseases are broadly categorized into three phenotypes: ulcerative colitis (UC), Crohn’s disease (CD) and IBD unspecified (IBDU). The term IBDU is applied when no differential diagnosis between UC and CD can be made (Park et al., 2020). These chronic and recurrent diseases afflict people of all ages due to modern lifestyles and the rise of the fast food trend, but they particularly impact people of working age and place a significant socio-economic burden on them. During the 1^st^ decade of the 21^st^ century, there is a rise in IBD cases in Asia, Africa and South America and the frequency is rising at a faster rate (CDC, 2020). Due to urbanization and westernization of developing countries, the prevalence rate of IBD is around 0.5-24.5 cases/100,000 persons/year for UC and 0.1-16 cases/100,000 persons/year for CD. Overall, the incidence for IBD is 396 cases per 100,000 persons annually (Khan et al., 2020). IBD greatly reduces the quality of life, but fortunately, the mortality rate is very low. Key risk factor for mortality is associated between inflammatory bowel disease pathogenesis towards colorectal cancer. Clinical profiles of CD and UC are quite different. CD may affect any part of the gastrointestinal tract (GIT) that can be complicated by intestinal obstructions, abscess formation and perforation, for which surgery is required while UC target colon with major symptoms including frequent diarrhea, bleeding from rectum, vomiting, abdominal pain, weight loss, fatigue, anemia and extra-intestinal manifestations (Larabi et al., 2020). Exact molecular biology and pathogenesis of UC is still unknown but exaggerated response of inflammatory mediators including cytokines and reactive oxygen mediators’ activation majorly initiate inflammatory pathways that are generally accepted as basic pathological process that contribute towards the pathogenesis of UC (Colombo et al., 2018). Reactive oxygen species (ROS) and reactive nitrogen species (RNS), two of these reactive oxygen mediators, are damaging to GIT, and the body's defense system keeps their concentrations in check to prevent any negative effects. The intracellular organelles mitochondria, endoplasmic reticulum, peroxisomes, nucleus, cytosol, and extracellular matrix are where ROS are mostly formed. Overproduction of ROS reduces adenosine triphosphate (ATP) synthesis and damages mitochondrial DNA, which causes cell death (Maurer et al., 2020). The endogenous defense system, comprised of enzymatic antioxidants like glutathione peroxidase (GPX-1), catalase (CAT) and superoxide dismutase (SOD), builds an antioxidant barrier in the mucosa of GIT and protects the tissue against oxidative damage. RNS mainly consist of nitric oxide (NO) that is reported to produce intracellular damage to protein and nucleic acids of cells hence upregulating the gene expression involved in innate and adaptive immune responses in GIT. Moreover, cytokines directly contribute to the destruction of mucosal tissue and are important in the development of UC's pathophysiology. By boosting ROS generation, which is crucial in the development of IBD, interleukins (IL-1 and IL-6) and tumor necrotic factor-alpha (TNF-α) lessen the intensity of inflammation (Lu and Zhao, 2020). Current therapeutic strategies for IBD include corticosteroids, aminosalicylates, anti-TNF-α agents and immunosuppressive drugs but these drugs are associated with serious adverse effects including kidney problems and hypersensitivity reactions (Minaiyan et al., 2014). Hence, there is need for more accessible and safe targeted therapies with enhanced potential to restore the altered immune functions and decrease the inflammation of GIT mucosa. Use of medicinal herbs and plants are increasing because of their easy availability, low cost and minimal adverse effects and several studies have confirmed their effectiveness against IBD (Wariss et al., 2014). 

*Indigofera argentea* Burm. f. (*I. argentea*) usually known as neel, hathio and jantar, belongs to the family Fabaceae and it is widely dispersed in the desert area of Pakistan, India and Iran. It was reported that *I. argentea* known as its Sanskrit name “Kala klitaka” and English name “Wild Indigo” is the plant of deep Cholistan desert that is traditionally used in inflammatory and gastric disorders. Plant decoction is beneficial in body pain and fever and to cure burning sensations in the body. Ahmad et al. verified ethnobotanical uses of whole plant of *I. argentea* in pain, malaria and gastric complaints (Ahmad et al., 2014). Moreover, medicinal uses of *I. argentea* have also been reported in ethno-veterinary practices to treat traumatic wounds and non-healing ulcers (Joshi and Nishteswar, 2014). In the scientific literature, *I. argentea* whole plant ethanolic extract has been reported for its antioxidant, anti-inflammatory, analgesic and antipyretic properties and previous phytochemical studies confirmed the presence of saponins, glycosides, alkaloids, flavonoids and tannins (Javed et al., 2020). Furthermore, an experimental study conducted by Haider and Imran confirmed its use in gastric disorders validating its spasmolytic and anti-diarrheal effects in rats (Haidar and Imran, 2020). The current study, therefore, intended to estimate the intestinal anti-inflammatory potential along with possible mechanisms of action in Swiss albino mice in order to provide scientific ground for its folkloric use in the traditional system of medicine. This is because there has been no reported scientific evidence on IBD, despite the reported traditional and biological activities and phytochemicals discussed for the crude extract of *Indigofera argentea* (Ia.Cr).

## Materials and Methods

### Collection of plant materials

Plant material were collected from their natural habitat in fresh form; *I. argentea* (whole plant) was collected from Deengarh area of Bahawalpur district (Cholistan desert), Pakistan. Plant sample was identified and authenticated from Dr. Abdul Hameed (Plant Taxonomist), and plant sample was stored in the Herbarium of department of Pharmacology, faculty of Pharmacy, the Islamia university of Bahawalpur, Pakistan and a voucher number was issued for the specimen submitted for future reference (*I. argentea*; IA-WP-09-14-76).

### Preparation of extract

Coarse grinding of plant material was done by using grinder to convert dried herbal stuff into coarse powder. Pulverized material of 1.5 kg was soaked in hydro-alcoholic mixture (30:70 v/v) at room temperature (25-30^°^C) for three days. Soaked material was stirred daily and subjected to straining via muslin cloth and further filtration through Whatman grade 1 filter paper after three days. Above process was repeated twice and resulting filtrates were mixed and solvents were evaporated in rotary evaporator at 37°C to get a greenish brown semisolid paste. The extract was weighed (to calculate percentage yield), labelled and stored in freezer at -20°C for future use.

### Drugs and reagents

Drugs and reagents used were Acetic acid (Fluka Analyticals, US), Formalin (Riedel-de Haen, Germany), Sulfasalazine (Ferozsons Laboratories, Pakistan), Potassium dihydrogen phosphate (BDH England), 0.9% Sodium chloride solution (Unisa Pharmaceuticals, Pakistan), Potassium chloride (Merck, Germany), Disodium hydrogen phosphate (Merck, Germany), Xylazine (Prix Pharmaceutical, Pakistan), and Ketamine (Global Pharmaceutical, Pakistan). 

### Preliminary phytochemical screening

Ia.Cr was screened for the detection of different phyto-constituents e.g. flavonoids, alkaloids, phenols, saponins, tannins, etc. (Shaikh and Patil, 2020).

### HPLC analysis

After making a stock solution using several standards, it was diluted with ethanol to achieve a 50 g/ml concentration. At a concentration of 10 mg/ml, ethanol was used as a dissolvable solvent for polyphenols. Fresh test samples were produced before being examined. For the analysis, a SIL-20A auto sampler and a C-18 column were employed. The linear gradient solution system had acetic acid, water, and ethanol flowing at a rate of 1 ml/min. The absorbance was measured at 280 nm, and the components were identified by comparing them to the reference solutions (Khan et al., 2017).

### LCMS analysis

As previously described by Khan et al., Ia.Cr was further processed for the identification of bioactive chemicals by LCMS analysis (Khan et al., 2017). The detection was carried out in positive-mode using a direct injection mode with an Electron Spray Ionization (ESI) probe. The sample flow rate was set to 8 L/min and the capillary temperature was fixed at 280°C. The mass range was chosen to be between 50 and 1000 m/z. During MS/MS, the collision induced dissociation energy (CID) was kept between 10 and 45, depending on the nature of the parent molecule ion. The mobile phase ratio of methanol and acetonitrile was 80:20 (v/v). By infusing the analytes and manually adjusting the parameters, the MS parameters for each compound were tuned to provide the best ionization, ion transfer, and optimum signal of both the precursor and fragment ions. For all of the analytes, the source parameters were the same.

### Experimental animals

Swiss albino mice (weighing 25–40 g) were housed in the animal house of the pharmacology department's research lab at the Islamia University of Bahawalpur in Pakistan. A total of six mice could fit in each of the polycarbonate cages (47x34x18 cm^3^) used to house all of the mice utilized in the study. Throughout the study, the typical housing parameters of humidity (50–55%), temperature (23–25℃), and exposure to a 12–12–hr light–dark cycle, were maintained. Animals were fed a regular food and given unlimited access to water. Under registration number 11-2020/PAEC, studies were carried out in accordance with the regulations established by the Pharmacy Animal Ethics Committee (PAEC).

### Acute toxicity analysis

Ia.Cr acute toxicity analysis was carried out in accordance with Organisation for Economic Co-operation and Development (OECD), 423, recommendations. Swiss albino mice, weighing 25 to 40 g, were divided into groups (n=5), acclimated to the laboratory setting, and given access to food and drink as needed before the toxicity protocol began. The control group received normal saline at a dose of 10 ml/kg. After a 12-hr fast, Ia.Cr was administered to different mouse groups at dosages of 300, 1000, 3000, and 5000 mg/kg. Mice were watched everyday for fourteen days starting on the first day at intervals of one hour. Animals were watched for signs of death or any behavioral changes, such as shaking, perspiring, convulsions, somatomotor activity, and changes in behavior pattern (Javed et al., 2020).

### Induction of ulcerative colitis

The procedure by Wang et al. was adopted with minor modifications (Wang et al., 2019). Mice were randomly divided into five groups (n=6). *Group I*: Normal Control, 0.9% normal saline (10 ml/kg, p.o). *Group II*: Intoxicated, 0.9% normal saline (10 ml/kg, p.o) + 200 μl of 7.5% of acetic acid (intra-rectal).* Group III*: Standard, Sulfasalazine (500 mg/kg p.o) + 200 μl of 7.5% of acetic acid (intra-rectal). *Group IV*: Treatment 1, *I. argentea* crude extract (300 mg/kg) + 200 μl of 7.5% of acetic acid (intra-rectal). *Group V*: Treatment 2, *I. argentea* crude extract (500 mg/kg) + 200 μl of 7.5% of acetic acid (intra-rectal)

All the agents were administered by gastric gavage once a day consecutively for seven days. On the eighth day, after fasting of 24 hr, UC was induced by intra-rectal administration of 7.5% of acetic acid at the dose of 200 μl. Mice were given a 10:1 combination of ketamine (50 mg/kg) and xylazine (5 mg/kg) at a dose of 0.2 ml per 100 g of animal body weight before receiving an equal volume of acetic acid through a Teflon catheter (0.8 mm in diameter) inserted 5–6 cm into the anus. The control group underwent a similar procedure but was given saline instead of acetic acid. To stop the implanted solution from escaping and ensure that acetic acid was distributed evenly throughout the colon, the mice were then placed in a supine Trendelenburg position for 30 sec. Animals were euthanized 24 hr later, and blood, spleen, and colonic tissues were taken from each animal. For histological examinations, pieces of colorectal tissues were kept in 10% formalin.

### Macroscopic parameters analysis

#### Disease activity index (DAI)

A clinical score that considered weight loss, rectal bleeding, and stool consistency, was used to evaluate DAI. Each score was calculated using the severity table provided by Niu et al. (Niu et al., 2013). Each parameter's score was summed together, divided by 3, and converted to a clinical score from 0 to 4 (Table 1). 

#### Colonic mucosal damage index (CMDI)

For each mouse, colon and rectum was separated and cut in longitudinal section, cleaned in saline to remove all the fecal material and weighed. Using the scoring pattern developed by Jagtap et al., macroscopic inflammation scores including gross lesion scores (Table 2) and percent area affected (Table 3) were assigned based on the clinical characteristics of the colon and rectal tissue. Colon and rectum scores were added together to determine a mouse's overall score (Jagtap et al., 2004).

After evaluating the parameters of DAI and CMDI, mice were sacrificed and their spleen and colonic tissues were separated to prepare proximal rectal tissue sections. Spleen and colonic tissue weight and length were measured for each animal for superficial assessment of the severity of inflammation (Thippeswamy et al., 2001).

### Hematological parameters analysis

At the end of the experiment, mice were beheaded, and blood was extracted and kept for hematological analysis at 2-8°C in ethylendiaminetetraacetic acid (EDTA)-covered test tubes.

### Determination of hematological parameters

The parameters investigated were white blood cell count (WBC), red blood cell count (RBC), hemoglobin concentration (Hb), hematocrit value (HCT) and platelet count (PLT) (Gupta et al., 2018). 

### Biochemical parameters analysis

For biochemical parameters assessment, animals were sacrificed under anesthesia (which was induced by using 10:1 combination of ketamine (50 mg/kg) and xylazine (5 mg/kg) at a dose of 0.2 ml per 100 g of animal body weight) and the colon sections were isolated and stored immediately at -80°C until further analysis. For further studies, 500 mg of colonic tissue sections was excised, rinsed, sliced and homogenized in ice cold phosphate buffer (pH 7.1) at a concentration of 10% w/v to obtain tissue homogenate. The supernatant obtained after centrifuging the tissue homogenate at 3,000 rpm (4°C) for 30 min, was used for biochemical analysis.

### Determination of colonic SOD, GPX-1 and CAT contents

The water-soluble Tetrazolium salt (WST-1) method was used to assess the SOD activity. The activity of SOD was determined by colorimetric measurement of the WST-1 product at OD of 450 nm after Xanthine Oxidase (XO) cleaved WST-1, which reacts with O_2_ to produce formazan dye while SOD activity negatively correlated with the amount of formazan dye. As a component of the glutathione system, GPX-1 uses selenium to catalyze the reduction of hydrogen peroxides with glutathione in order to protect cells from oxidative damage. According to the manufacturer's instructions, a commercial ELISA kit was used to measure the level of GPX-1, an antioxidant enzyme (Gupta et al., 2018). The most significant hydrogen peroxide (H_2_O_2_) specie scavenger CAT is widely distributed in animals, plants, microbes, and culture cells and plays a significant role in the scavenging system of reactive oxygen species. H_2_O_2_ has specific absorbance peak at 240 nm. CAT can decompose H_2_O_2 _and the absorbance value of 240 nm will decrease as reaction goes on. CAT activity can be calculated according to the changing rate of absorbance value (Ryan et al., 2020).

### Determination of lipid peroxidation activity by malondialdehyde (MDA) assay and nitric oxide assay

MDA interacts with thiobarbituric acid (TBA) to produce the MDA-TBA adduct in the lipid peroxidation by malondialdehyde assay. The amount of MDA-TBA adduct was calculated to be at an OD of 450 nm . The Griess method was used to assess the quantity of NO in the colon, and a colorimetric assay kit was used to measure absorbance at 550 nm (Gupta et al., 2018). 

### Determination of colonic cytokines (IL-1β, IL-6 and TNF-α) level

The cytokines immunoassay kit was used to perform the quantifications of IL-1, IL-6, and TNF-α. Sandwich enzyme immunoassay was used in the experiment. Microtiter plates for ELISA kits come pre-coated with particular antibodies to IL-1, IL-6, and TNF-α. Standard solutions and samples were added to the plate wells and incubated at 37℃ for 2 hr. Plates were washed and horseradish peroxidase (HRP) conjugate solutions of IL-1β, IL-6 and TNF-α were added to each well and incubated at 37℃ for 1 hr. Wash the plates again and add tetramethylbenzidine (TMB) substrate stop solution to stop the reaction after incubation at 37℃ for 15 min and read the plate at an optical density of 450 nm (Wang et al., 2019; Ryan et al., 2020). 

### Microscopic parameter analysis (histological examination)

Selecting one mouse from each group, the colon tissue was excised, cleansed with saline, and preserved in 10% formalin for microscopic histological examination. For a microscopic examination, it was further treated for 12 hr with xylene, isopropyl alcohol, and paraffin. For morphological evaluation of colon damage, tissue sections embedded in paraffin were cut into 5 µm-thick slices, deparaffinized, and stained with hematoxylin and eosin stain (H&E). Photomicrographs at a 10x magnification were taken (Wang et al., 2019).

### Statistical analysis

The findings are presented as mean S.E.M. Using the program GraphPad Prism 8.0, data analysis was carried out (GraphPad, SanDiego, CA). Statistics were compared between the treatment groups and the intoxicated group as well as between the normal control and the intoxicated group. One-way analysis of variance was used to assess the data for macroscopic, biochemical, and hematological parameters, and Tukey Kramer's multiple comparison test was used for post hoc analysis. A p value of 0.05 or lower was regarded as statistically significant.

## Results

### Preliminary phytochemical screening

Ia.Cr (with percent yield of 13.2%) was screened for different phytochemical constituents. Phytochemical testing revealed the presence of medicinal important secondary metabolites including saponins, steroids, alkaloids, flavonoids, phenols, and tannins.

### HPLC analysis

HPLC fingerprinting of Ia.Cr revealed the presence of gallic acid, quercetin, benzoic acid, chlorogenic acid, caffeic acid, ferulic acid and p-coumaric acid (Table 4).

### LCMS analysis

LCMS analysis revealed the presence of various phytoconstituents summarized in Table 5.

### Acute toxicity analysis

Ia.Cr was tested following OECD guidelines at different doses and found safe up to the maximum dose of 5000 mg/kg. 

### Macroscopic analysis of acetic acid-induced ulcerative colitis

#### Disease activity index (DAI)

Mice treated with acetic acid showed diarrhea, rectal bleeding, hypomotality with major reduction in body weight at the 9^th^ day when compared with the first day. Pre-treatment with Ia.Cr (300 and 500 mg/kg, p.o) for seven days considerably helped normalize the parameters. Sulfasalazine (500 mg/kg, p.o) also exhibited significant decline in disease activity index when compared to the acetic acid intoxicated group (Table 6). 

### Colonic mucosal damage index (CMDI)

Colonic mucosal damage score described macroscopic evidence of widespread injury of colonic mucosa after 24 hr of colitis induction. Mucosa appeared edematous, hemorrhagic and ulcerated as compared to the normal control group. Pre-treatment with Ia.Cr for seven days significantly decreased the gross lesion severity score as compared to the intoxicated group. Standard treatment sulfasalazine also produced a significant drop in macroscopic score as compared to the intoxicated group (Table 6).

### Effect of Ia.Cr on wet weight of colon

As shown in Table 3, wet weight of colon tissue of intoxicated group was much higher (1268±8.92 mg) when compared to the normal control group (741.7±13.02 mg) that serves as the hallmark of edema. In Ia.Cr-treated groups (300 and 500 mg/kg p.o), a significant reduction in the wet weight of colon was observed as well as in mice treated with sulfasalazine. Results are summarized in Table 6.

### Effect of Ia.Cr on colon weight to length ratio

Acetic acid-induced intoxicated group showed substantial increase in the colon weight to length ratio (206.4±6.58 mg/cm) in comparison to the normal control group (73.97±1.62 mg/cm) while Ia.Cr (300 and 500 mg/kg p.o) and sulfasalazine mitigated the weight to length ratio of the mice colon in comparison with the intoxicated group (Table 6).

### Effect of Ia.Cr on spleen weight

Mice in the acetic acid-induced intoxication group showed splenic enlargement in comparison with the normal control group. Treatment with sulfasalazine and Ia.Cr significantly (p˂0.001) attenuated this augmented spleen weight as compared to the intoxicated group (Table 6).

### Hematological parameters analysis

Hematological parameters WBC, RBC, PLT, Hb, and HCT showed a significant decrease in acetic acid-treated mice as compared to the normal healthy mice. This decrease in the levels of hematological parameters was attenuated significantly (p˂0.001) in Ia.Cr pre-treated groups as compared to the acetic acid-treated group. Results are summarized in Table 7.

### Biochemical parameters analysis

#### Determination of colonic SOD, GPX-1 and CAT contents

**Figure 1:**
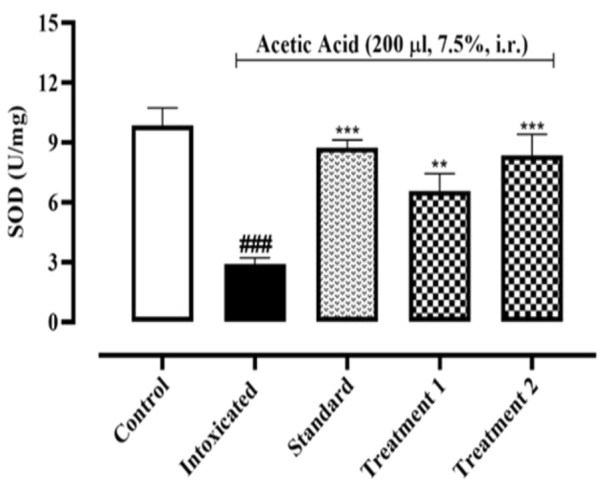
Effects of different doses of Indigofera argentea (treatment group 1 and 2 at the doses of 300 and 500 mg/kg, respectively) and sulfasalazine (the standard group at the dose of 500 mg/kg) on antioxidant enzyme system. Control group received normal saline while intoxaication was done by administering 7.5% acetic acid at the dose of 200µl via intrarectal (i.r.) route. Three independent experiments were performed and colonic level of SOD was measured calorimetrically. Data are expressed as mean±SEM; ###p˂0.001 vs the normal control; and *p˂0.05, **p˂0.01 and ***p˂0.001 vs the intoxicated group.

**Figure 2:**
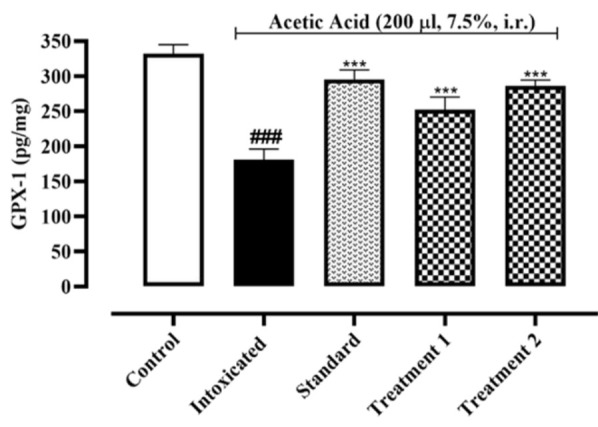
Effects of different doses of Indigofera argentea (treatment group 1 and 2 at the doses of 300 and 500 mg/kg, respectively) and sulfasalazine (the standard group at the dose of 500 mg/kg) on antioxidant enzyme system. Control group received normal saline while intoxaication was done by administering 7.5% acetic acid at the dose of 200µl via intrarectal (i.r.) route. Three independent experiments were performed and colonic level of GPX-1 was measured calorimetrically. Data are expressed as mean±SEM; ###p˂0.001 vs the normal control; and *p˂0.05, **p˂0.01 and ***p˂0.001 vs the intoxicated group.

As shown in Figure 1, the concentration of SOD enzyme decreased significantly in the colonic tissue of the intoxicated group when compared to the normal control group. On the other hand, Ia.Cr treatments at 300 and 500 mg/kg increased enzyme concentration (p˂0.05). As depicted in Figure 2, acetic acid-induced colitis produced a significant decrease in GPX-1 contents in the colonic tissues of the intoxicated group (181.1±8.8 pg/mg of protein), when compared with that of the normal control group (332.2±7.38 pg/mg of protein). Also, 7 days of pre-treatment with Ia.Cr (300 and 500 mg/kg) significantly increased GPX-1 contents (252.2±10.4 and 286.1±4.85 pg/mg of protein respectively) as compared with the intoxicated group. Sulfasalazine also showed a significant protection against GPX-1 depletion induced by acetic acid (295.5±7.68 pg/mg of protein).

**Figure 3:**
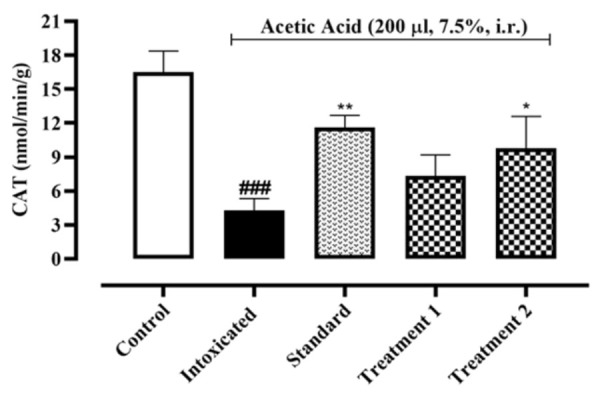
Effects of different doses of Indigofera argentea (treatment group 1 and 2 at the doses of 300 and 500 mg/kg, respectively) and sulfasalazine (the standard group at the dose of 500 mg/kg) on antioxidant enzyme system. Control group received normal saline while intoxaication was done by administering 7.5% acetic acid at the dose of 200µl via intrarectal (i.r.) route. Three independent experiments were performed and colonic level of CAT was measured calorimetrically. Data are expressed as mean±SEM; ###p˂0.001 vs the normal control; and *p˂0.05, **p˂0.01 and ***p˂0.001 vs the intoxicated group.

Development of acetic acid-induced colitis decreased CAT level in ulcerated tissues of the intoxicated group (4.28±0.61 nmol/min/g) when compared to the normal control group (16.52±1.10 nmol/min/g). Pre-treatment with Ia.Cr 300 and 500 mg/kg increased CAT level to 7.34±1.05 and 9.79±1.62 nmol/min/g respectively and showed significant antioxidant activity when compared to the intoxicated group (Figure 3). 

### Determination of lipid peroxidation activity by malondialdehyde assay and nitric oxide assay

**Figure 4:**
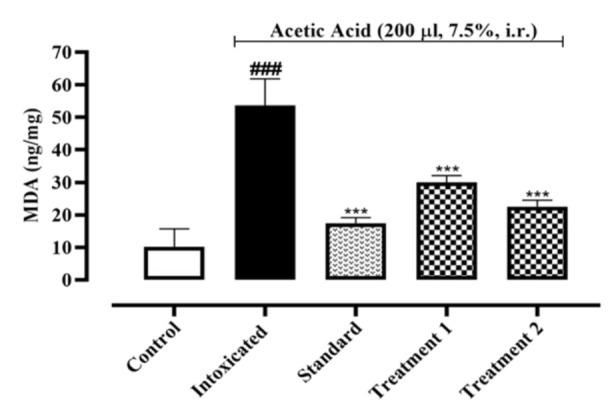
Effects of different doses of Indigofera argentea (treatment group 1 and 2 at the doses of 300 and 500 mg/kg, respectively) and sulfasalazine (the standard group at the dose of 500 mg/kg) on lipid peroxidation level, measured as the concentration of MDA. Control group received normal saline while intoxaication was done by administering 7.5% acetic acid at the dose of 200µl via intrarectal (i.r.) route. Three independent experiments were performed and colonic level of MDA was measured by ELISA assay. Data are expressed as mean±SEM; ###p˂0.001 vs the normal control; and *p˂0.05, **p˂0.01 and ***p˂0.001 vs the intoxicated group.

Estimation of lipid peroxidation in colon tissue of acetic acid-induced ulcerative colitis was performed by MDA-TBA method. Animals in the intoxicated group, treated with acetic acid, resulted in increased lipid peroxidation, described by an increase in MDA level (53.64±4.71 ng/mg of protein) as compared to the normal control group (10.19±3.18 ng/mg of protein). Sulfasalazine considerably reduced the elevated MDA level in tissue, bringing it down to 17.50 0.96 ng/mg of protein. When compared to the intoxicated group, both doses (300 and 500 mg/kg) evaluated for Ia.Cr reduced lipid peroxidation at levels of 30±1.20 and 22.53±1.12 ng/mg of protein, respectively (Figure 4).

**Figure 5:**
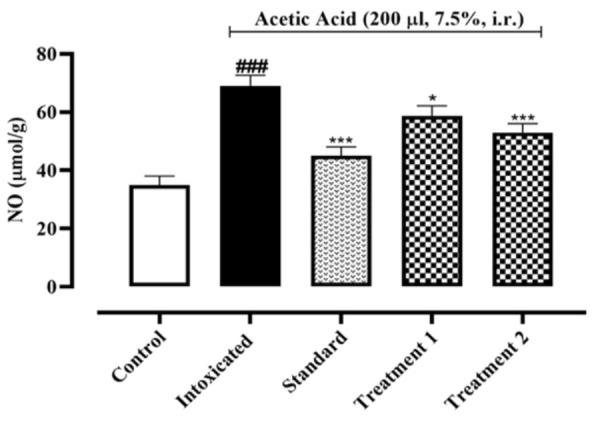
Effects of different doses of Indigofera argentea (treatment group 1 and 2 at the doses of 300 and 500 mg/kg, respectively) and sulfasalazine (the standard group at the dose of 500 mg/kg) on Nitric oxide assay. Control group received normal saline while intoxaication was done by administering 7.5% acetic acid at the dose of 200µl via intrarectal (i.r.) route. Two independent experiments were performed and colonic level of NO was measured calorimetrically. Data are expressed as mean±SEM; ###p˂0.001 vs the normal control; and *p˂0.05, **p˂0.01 and ***p˂0.001 vs the intoxicated group.

When compared to the control group, the level of NO in colon tissue increased significantly (p˂0.001) in the intoxicated group. Sulfasalazine and Ia.Cr treatment significantly lowered elevated NO levels (p˂0.001) when compared to the intoxicated group (Figure 5). 

### Determination of colonic cytokines (IL-1β, IL-6 and TNF-α) level

**Figure 6:**
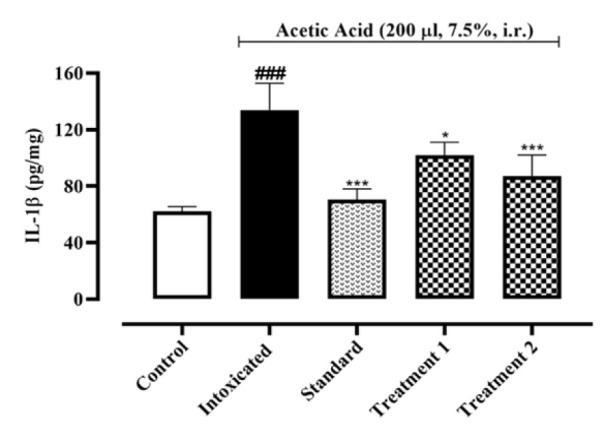
Effects of different doses of Indigofera argentea (treatment group 1 and 2 at the doses of 300 and 500 mg/kg, respectively) and sulfasalazine (the standard group at the dose of 500 mg/kg) on the levels of IL-1β. Control group received normal saline while intoxaication was done by administering 7.5% acetic acid at the dose of 200µl via intrarectal (i.r.) route. Three independent experiments were performed and colonic levels of IL-1β were measured using sandwich ELISA assay. Data are expressed as mean±SEM; ###p˂0.001 vs the normal control; and *p˂0.05, **p˂0.01 and ***p˂0.001 vs the intoxicated group.

**Figure 7:**
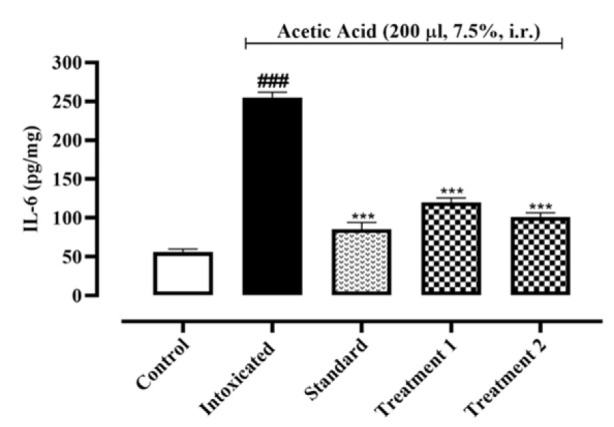
Effects of different doses of Indigofera argentea (treatment group 1 and 2 at the doses of 300 and 500 mg/kg, respectively) and sulfasalazine (the standard group at the dose of 500 mg/kg) on the levels of IL-6. Control group received normal saline while intoxaication was done by administering 7.5% acetic acid at the dose of 200µl via intrarectal (i.r.) route. Three independent experiments were performed and colonic levels of IL-6 were measured using sandwich ELISA assay. Data are expressed as mean±SEM; ###p˂0.001 vs the normal control; and *p˂0.05, **p˂0.01 and ***p˂0.001 vs the intoxicated group.

**Figure 8:**
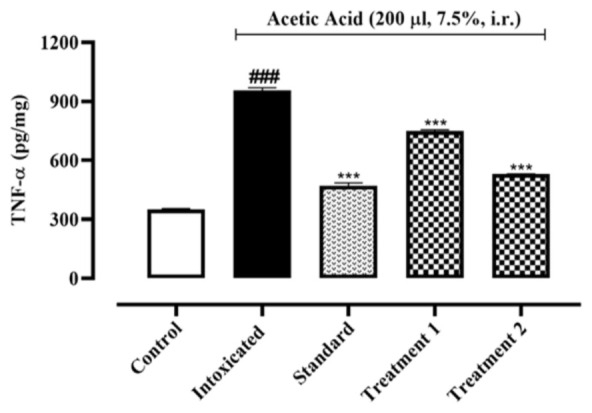
Effects of different doses of Indigofera argentea (treatment group 1 and 2 at the doses of 300 and 500 mg/kg, respectively) and sulfasalazine (the standard group at the dose of 500 mg/kg) on the levels of TNF-α. Control group received normal saline while intoxaication was done by administering 7.5% acetic acid at the dose of 200µl via intrarectal (i.r.) route. Three independent experiments were performed and colonic levels of TNF-α were measured using sandwich ELISA assay. Data are expressed as mean±SEM; ###p˂0.001 vs the normal control; and *p˂0.05, **p˂0.01 and ***p˂0.001 vs the intoxicated group.

When compared to the normal control group (62.25±1.96, 56.31±2.01, 351.9±1.93 pg/mg of protein), acetic acid intoxication led to a rise in IL-1, IL-6, and TNF-α by 133.9±10.93, 254.9±3.99, and 956.4±7.57 pg/mg of protein, respectively. Pre-treatment with Ia.Cr (300 and 500 mg/kg) reduced IL-1β and IL-6 concentrations 101.8±5.32, 120.2±3.12 and 87.25±8.51, 101.3±3.12 pg/mg of protein respectively (p˂0.05), while sulfasalazine attained reduction in IL-1β and IL-6 concentrations to 70.58±4.41 and 85.42±5.02 pg/mg of protein when compared with the control group (Figure 6, 7). Treatment with Ia.Cr also decreased TNF-α concentration at all tested doses by 749.6±3.53 and 530.1±0.84 pg/mg of protein respectively. In the similar way, sulfasalazine also reduced TNF-α concentration to 471.7±8.03 pg/mg of protein when compared to the intoxicated group (Figure 8). 

### Microscopic parameter analysis (Histological examination)

Histopathology of H&E-stained colons specimen showed immense necrotic damage to epithelium, edema of sub-mucosa, hemorrhage and infiltration of inflammatory cells in the mucosa of intestinal tissue. Acetic acid-treated mice showed massive production of inflammation that was extended to muscularis and sub-mucosal layer with extensive tissue granulation and lymphocyte and fibroblast diffusion. Ulcerated mucosa showed crypt distortion and disruption of epithelium and loss of goblet cells were visible in surface epithelium of intoxicated tissue (Figure 4B) compared to normal tissue sample (Figure 4A). Treatment with sulfasalazine (Figure 4C) and *I. argentea* crude extract (Figures 4D and 4E) significantly reduced the severity and extent of tissue damage. Mucosal tissue showed lesions in healing process and mononuclear dominance with initiation of repair process.

## Discussion

Current study was specifically designed to explore the potential of *I. argentea* crude extract against acetic acid-induced ulcerative colitis in mice, as this model is extensively accepted to monitor the potential of different agents for preclinical testing against ulcerative colitis because of its resemblance to the inflammatory bowel disease in humans including transmural inflammation of colon, neutrophil infiltration of mucosa, mucosal inflammation along sub-mucosal ulceration, scarring, fibrosis of tissue, fecal impaction, diarrhea, stenosis and oxidative stress (Jagtap et al., 2004). Acetic acid is believed to produce intestinal inflammation by damaging epithelium due to intracellular acidification and liberation of protons from acid that results in epithelial injury of intestinal tract. Initial injury was mild initiating damage to colon by enhancing vaso-permeability, necrosis of epithelium that extended deep into the lamina propria, sub-mucosa or external layers of muscle, depending on the length of exposure and concentration of acetic acid (Niu et al., 2013). Damage to mucosa and sub-mucosa produce local immune response with infiltration of macrophages and lymphocytes followed by the liberation of reactive oxygen metabolites (ROS and RNS), cytokines and other mediators resulting in lipids, proteins and nucleic acid cross-linking which cause cellular dysfunction and intestinal tissue damage (Ryan et al., 2020). Antioxidant enzymes SOD, GPX, and CAT, NO and release of pro-inflammatory cytokines play a significant role in the progression of UC. IL-1β, IL-6 and TNF-α enhance the production of ROS and activate oxidative stress responsive genes which in turn amplify and exacerbate the inflammation (Arunachalam et al., 2020). Acetic acid-induced UC was found to be associated with macroscopic, hematological, biochemical, and microscopic alterations in the current investigation. Hallmarks of pathological process of IBD majorly include destruction of mucosal barrier and colon structure, increased vaso-permeability, release of inflammatory mediators and tissue fibroelastic core process disturbance with fibrin hydrolysis (Shahrokhi et al., 2018). In this study, pre-treatment with *I. argentea *crude extract significantly decreased the DAI, CMDI, wet weight of colon, spleen weight, wet colon weight/length ratio and macroscopic score, when compared with the intoxicated group, representing its protective anti-inflammatory effect against UC. Analysis of hematological parameters is crucial for pinpointing the anemia and inflammation that characterize IBD. RBC count is significant because hemoglobin found in RBCs is used to carry oxygen throughout the body. Anemia, a common symptom of IBD caused by poor iron absorption and persistent intestinal bleeding, is indicated by a low RBC count. A condition known as anemia of chronic disease (ACD) or anemia of inflammation is indicated if both RBC and Hb levels are low. ACD is the defining characteristic of diseases of chronic inflammation, such as IBD (Ahluwalia et al., 2018). WBCs are also an important component of the immune system. Low WBCs show the blunt response of immune system to the infectious diseases indicating the state of high risk of infection (Olamilosoye et al., 2018). Moreover, decrease in WBC count could be due to the effect of immunosuppressive actions on bone marrow by inhibiting the activity of T cells (Zenlea and Peppercorn, 2014). *Indigofera argentea* crude extract, at the doses of 300 and 500 mg/kg, significantly normalized the hematological parameters that could be due to the presence of constituents that stimulate bone marrow to produce blood cells and release them in the blood. 

Recent studies showed that oxidative stress aggravates IBD severity (Ryan et al., 2020). Ia.Cr decreased the MDA levels significantly in the colon tissues. SOD, GPX-1, and CAT are additional significant reactive oxygen metabolites that have a role in the pathophysiology of colitis. SOD is an enzyme that converts O_2_^˚^ to O_2_ and H_2_O with the help of a metal ion cofactor. CAT and GPX-1 combine to produce H_2_O_2_ as a byproduct. To keep the ROS constant state, these defense enzymes collaborated in a balanced and coordinated manner. In our study, administration of Ia.Cr resulted in significant increase in SOD, GPX-1 and CAT activities when compared with the colitis-induced group. Thus, the protective effects of Ia.Cr may be attributed to the direct free radical scavenging and antioxidant activities as well as the RNS scavenging effects (Hur et al., 2012). UC is an inflammatory disease that majorly targets the mucosa and sub-mucosa of the colon. Exact pathogenic mechanism of UC is still unclear and researchers support the hypothesis that increase surge of pro-inflammatory mediators like TNF-α and interleukins (IL-1β and IL-6) contribute in the inflammation cascade resulting in pathological progression of disease and severe tissue damage (Wang et al., 2019). TNF-α promotes the expression of chemokines by an induction molecule, which causes a significant inflow of inflammatory cells. As a result, TNF- α is now a preferred target for many therapeutic drugs used to treat IBD (Arunachalam et al., 2020). While IL-1 plays a significant role in inflammation and innate immunity, which contribute to tissue damage in IBD, IL-6 is an essential cytokine in the pathogenesis of UC since it boosts neutrophil chemotaxis to promote necrosis in the colon and promote tissue death. Levels of IL-1 directly correlate positively with the degree of mucosal inflammation. Epithelial cell death is boosted by IL-1, which damages tissue and interferes with barrier function (Adjouzem et al., 2020). Moreover, treatment with Ia.Cr significantly decreased the levels of TNF-α, IL-6 and IL-1β in colon, which indicate the beneficial effects of Ia.Cr against intestinal inflammation. In the current study, sulfasalazine was used as standard treatment for IBD. Ia.Cr (300 and 500 mg/kg) showed significant anti-inflammatory effects in experimental IBD and sulfasalazine at the dose of 500 mg/kg, was superior in treating IBD. The findings of the medications employed to treat UC were supported by the histology of colon tissues. Microscopic examination revealed goblet cell loss, bleeding in the crypts, and neutrophil and lymphocyte infiltration in sub-mucosa in a group of mice that had consumed alcohol (Wang et al., 2019). Infiltration of neutrophils in the inflamed mucosa is the major microscopic feature detected in IBD and these activated neutrophils produce ROS/RNS within mucosa to induce oxidative stress that significantly contribute in the pathogenesis of IBD in animals and humans (Arunachalam et al., 2020). Sulfasalazine and Ia.Cr decreased neutrophil infiltration resulting in a decline of damage to intestinal mucosa.

Preliminary phytochemical screening of *I. argentea* ethanolic crude extract revealed the presence of phenols, flavonoids, saponins, alkaloids, steroids and tannins. Quercetin, gallic acid, caffeic acid, chlorogenic acid, benzoic acid, ferulic acid, and p-coumaric acid were all found in Ia.Cr after being subjected to HPLC and LCMS analysis. According to reports, quercetin possesses potent anti-inflammatory and antioxidant properties (Javed et al., 2020). According to studies, coumaric acid also demonstrated anti-inflammatory activity by decreasing the release of cytokines to prevent tissue damage by scavenging ROS, while gallic acid is known to have potent antioxidant and anti-inflammatory potential and induce apoptosis in colon adenocarcinoma in cell lines, delaying the malignancy and developmental progression of cancer (Javed et al., 2020). Kaempferide has significant anti-inflammatory and hypoglycemic properties. The mechanism may be linked to the TLR4/IκBα/NF-κB signaling pathway, which modulate the inflammatory process and oxidative damage (Tang et al., 2021). Hence, taken together all the findings of the study, it could be proposed that whole plant of *I. argentea* is a promising traditional medicine that can be used in inflammatory bowel disease in future.

The results of the current study suggest that Ia.Cr mitigated the acetic acid-induced IBD in mice by producing anti-inflammatory actions through the modulation of antioxidant enzyme and pro-inflammatory cytokines. Histological findings showed reduced hyperemia and ulceration revealing healing potential of *I. argentea* by decreasing inflammatory mediators, hence, multi-target mechanisms might be involved in the intestinal anti-inflammatory effects. Therefore, there is a need of further studies to evaluate the alternative and additional mechanisms involved in the management of inflammatory bowel disease.

Further studies to evaluate the efficacy of the crude extract of *I. argentea* against inflammatory and gastric disorders targeting anti-inflammatory cytokines and NF-kB pathway studies are warranted. Furthermore, molecular interactions responsible for biological activities also need to be explored.

**Table 1:** The scoring system for the evaluation of disease activity index (DAI)

Score	Weight Loss (%)	Stool Consistency	Rectal Bleeding
0	None 1-5	Normal	Normal
1			
2	5-10 10-20	Loose Stool	Bleeding
3			
4	˃20	Diarrhea	Gross Bleeding

(Niu et al., 2013)

**Table 2:** The scoring system for inflammatory macroscopic changes

Score	Inflammatory Macroscopic Changes
**0**	No visible change
**1**	Hyperemia at sites
**2**	Lesions having diameter (1mm or less)
**3**	Lesions having 2mm diameter (˂ 5 in no.)
**4**	Lesions having 2mm diameter (5-10 in no.)
**5**	Lesions having 2mm diameter (˃ 10 in no.)
**6**	Lesions having more than 2mm diameter (˂ 5 in no.)
**7**	Lesions having more than 2mm diameter (5-10 in no.)
**8**	Lesions having more than 2mm diameter (˃ 10 in no.)

(Jagtap et al., 2004)

**Table 3:** The scoring system for percent area affected

Score	Percent area affected
**0**	0
**1**	1-5
**2**	5-10
**3**	10-25
**4**	25-50
**5**	50-75
**6**	75-100

(Jagtap et al., 2004)

**Table 4:** Identified compounds from the crude extract of *Indigofera argentea *(Ia.Cr) by HPLC analysis

**Retention Time (min)**	**Compounds Identified**
2.693	Quercetin
4.33	Gallic acid
12.05	Caffeic acid
15.17	Benzoic acid
21.86	Ferulic acid
20.57	p-Coumaric acid

**Table 5:** Identified compounds from the crude extract of *Indigofera argentea *(Ia.Cr) by LCMS analysis

S. No.	RT	Name	Mass	Formula
**1**	0.638	N-Glycosyl-L-Asparagine	294.1	C_10_H_18_N_2_O_8_
**2**	0.657	Quebrachitol	194	C_7_H_14_O_6_
**3**	0.682	Quinic Acid	192	C_7_H_12_O_6_
**4**	0.712	Benzoic Acid	122	C_7_H_6_O_2_
**5**	0.885	Β-Hydroxypyruvic Acid	104	C_3_H_4_O_4_
**6**	0.987	Citric Acid	192	C_6_H_8_O_7_
**7**	7.231	p-Salicylic Acid	138	C_7_H_6_O_3_
**8**	8.404	Kaempferol 3-(2G-Glucosylrutinoside)	756.2	C_33_H_40_O_20_
**9**	8.685	Robinetin 3- Rutinoside	610.15	C_27_H_30_O_16_
**10**	9.356	2-methoxy-4-vinylphenol	150	C_9_H_10_O_2_
**11**	9.087	Hyperin	464.09	C_21_H_20_O_12_
**12**	9.090	Viscidulin I	302.04	C_15_H_10_O_7_
**13**	9.1	Azaleatin 3-galactoside	478.1	C_22_H_22_O_12_
**14**	9.295	Tricetin 7-methyl ether 3’-glucoside-5’-rhamnoside	624.16	C_28_H_32_O_16_
**15**	9.559	6-Hydroxyluteolin 4’-methyl ether	316	C_37_H_62_N_10_O_2_
**16**	9.588	2,6-dimethoxyphenol	154	C_8_H_10_O_3_
**17**	9.765	Kaempferide	300.06	C_16_H_12_O_6_
**18**	9.768	Syringin	372.14	C_17_H_24_O_9_
**19**	10.181	Vanillin (4-hydroxy-3-methoxybenzaldehyde)	152	C_8_H_8_O_3_
**20**	11.445	Quillaic acid 3-rhamnosyl-galactosyl-glucuronide	970.47	C_48_H_74_O_20_
**21**	10.478	Luteolin 5, 3-dimethyl ether	314	C_17_H_14_O_6_
**22**	10.72	5,7,2,3-Tetrahydroxyflavone	286	C_15_H_10_O_6_
**23**	10.787	Melanoxetin	302.04	C_15_H_10_O_7_
**24**	11.004	Nodifloretin	316.05	C_16_H_12_O_7_
**25**	11.027	Syringetin	346	C_17_H_14_O_8_
**26**	11.963	Chicoric acid	474.07	C_22_H_18_O_12_
**27**	12.260	Jujubasaponin IV	942.52	C_48_H_78_O_18_
**28**	12.271	Calenduloside A	780.46	C_42_H_68_O_13_
**29**	13.838	Morphine	285.13	C_17_H_19_NO_3_
**30**	12.866	Dehydrosoyasaponin I	940.5	C_48_H_76_O_18_
**31**	13.437	Gingerol	294.18	C_17_H_26_O_4_
**32**	14.045	13-cis-retinal	284.2	C_20_H_28_O
**33**	14.048	Palmyrolide A	337.26	C_20_H_35_NO_3_
**34**	14.565	Bufotalin	444.25	C_26_H_36_O_6_
**35**	17.134	Emmotin A	278.15	C_16_H_22_O_4_
**36**	20.597	Pheophorbide A	592.26	C_35_H_36_N_4_O_5_
**37**	22.054	Stearic Acid	284.5	C_18_H_36_O_2_
**38**	24.935	3α, 12α-Dihydroxy-5β-chol-8(14)-en-24-oic Acid	390.27	C_24_H_38_O_4_

**Table 6:** Effects of the crude extract of *Indigofera argentea* (Ia.Cr) on disease activity index (DAI), colonic mucosal damage index (CMDI), spleen weight, colon weight and colon weight/length ratio against acetic acid-induced inflammatory bowel disease in mice

**Treatment**	**DAI** **(% Protection)**	**CMDI** **(% Protection)**	**Spleen Weight** **(mg)**	**Colon Weight** **(mg)**	**Colon Weight/Length** **(mg/cm)**
Normal Control (NS 10ml/kg)	0	0	215± 0.02	741.7 ± 13.01	73.97 ± 1.62
Intoxicated (AA 200µl of 7.5%)	3.50±0.50^###^	6.3±0.7^###^	410 ± 0.02^###^	1268 ± 8.92^###^	206.4 ± 6.58^###^
Sulfasalazine (500mg/kg)	0.65±0.18^***^ (81.4%)	2.9±0.1^**^ (53.9%)	254 ± 0.01^***^	876.7 ± 14.76^***^	94.49 ± 2.13^***^
Ia.Cr (300 mg/kg)	1.16±0.58^**^ (66.8%)	4.9±0.4 (22.2%)	298 ± 0.01^***^	1148 ± 14.93^*** ^	145.3 ± 3.25^***^
Ia.Cr (500 mg/kg)	0.72±0.36^***^ (79.4%)	3.8±0.2^*^ (39.7%)	282 ± 0.01^***^	950 ± 9.31^***^	112.3 ± 1.86^***^

Values are given as mean±SEM of 6 animals in each group, one-way ANOVA Tukey-Kramers; values are statistically significant at ^###^p˂0.001, between normal and intoxicated group, ^***^p˂0.001, ^**^p˂0.01 and ^*^p˂0.05 between intoxicated and treated groups.

**Table 7:** Effects of the crude extract of *Indigofera argentea* (Ia.Cr) on hematological parameters against acetic acid-induced inflammatory bowel disease in mice

Treatment	WBC (x10^3^/µl)	RBC (x10^6^/µl)	Hb (g/dl)	PLT (x10^5^/µl)	HCT (%)
**Normal Control (NS 10ml/kg)**	16.75 ± 0.16	16.10 ± 0.21	15.35 ± 0.28	10.95 ± 0.22	45.33 ± 1.54
**Intoxicated (AA 200µl of 7.5%)**	5.82 ± 0.19^###^	7.00 ± 0.16^###^	9.78 ± 0.24^###^	4.87 ± 0.18^###^	30.87 ± 0.46^###^
**Sulfasalazine (500mg/kg)**	15.42 ± 0.16^***^	14.25 ± 0.14^***^	13.80 ± 0.31^***^	9.93 ± 0.10^***^	42.02 ± 1.40^***^
**Ia.Cr (300 mg/kg)**	13.90 ± 0.12^***^	13.87 ± 0.19^***^	11.50 ± 0.24^***^	9.05 ± 0.09^***^	36.83 ± 1.01^*^
**Ia.Cr (500 mg/kg)**	14.43 ± 0.11^***^	14.23 ± 0.22^***^	12.68 ± 0.22^***^	9.62 ± 0.10^***^	39.83 ± 1.14^***^

Values are given as mean±SEM of 6 animals in each group, one-way ANOVA Tukey-Kramers; values are statistically significant at ^###^p˂0.001, between normal and intoxicated group, ^***^p˂0.001, ^**^p˂0.01 and ^*^p˂0.05 between intoxicated and treated groups.

**Figure 9:**
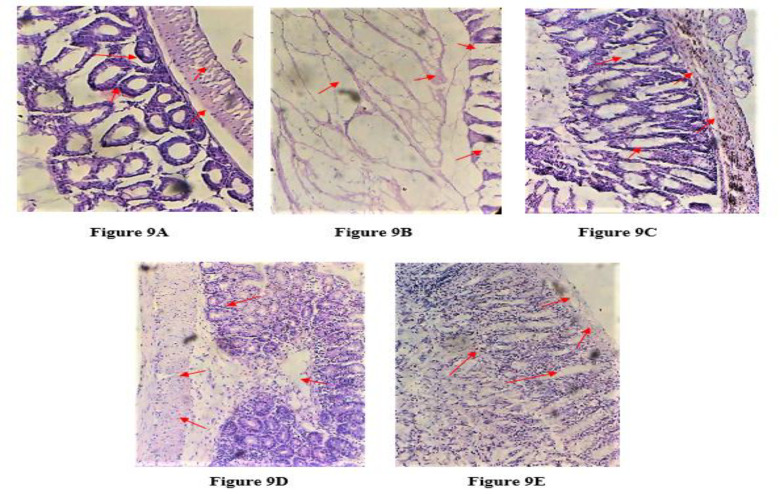
Pre-treatment with *Indigofera argentea* reduced histological alterations-induced by acetic acid (7.5%) in mice colon. Images were captured using a 10-fold magnification of colon tissue slices stained with hematoxylin and eosin. Colon microscopic image of (A) Normal mice with intact epithelial and mucosal layer; (B) Acetic acid-induced colitis mice with extensive damage to epithelial and mucosal layer and necrosis. (C) Sulfasalazine-treated mice, (D) *Indigofera argentea* (300 mg/kg, p.o.) and (E) *Indigofera argentea* (500 mg/kg, p.o.) 7 day pre-treated mice with decreased ulceration, necrosis and edema as compared to acetic acid-induced colitis mice.
